# Comparisons of *Shewanella* strains based on genome annotations, modeling, and experiments

**DOI:** 10.1186/1752-0509-8-31

**Published:** 2014-03-12

**Authors:** Wai Kit Ong, Trang T Vu, Klaus N Lovendahl, Jenna M Llull, Margrethe H Serres, Margaret F Romine, Jennifer L Reed

**Affiliations:** 1 University of Wisconsin-Madison, Madison, USA; 2 Marine Biological Laboratory, Woods Hole, USA; 3 Pacific Northwest National Laboratory, Richland, USA

**Keywords:** Constraint-based model, Electron acceptors, Phenotype, FBA

## Abstract

**Background:**

*Shewanella* is a genus of facultatively anaerobic, Gram-negative bacteria that have highly adaptable metabolism which allows them to thrive in diverse environments. This quality makes them an attractive bacterial target for research in bioremediation and microbial fuel cell applications. Constraint-based modeling is a useful tool for helping researchers gain insights into the metabolic capabilities of these bacteria. However, *Shewanella oneidensis* MR-1 is the only strain with a genome-scale metabolic model constructed out of 21 sequenced *Shewanella* strains.

**Results:**

In this work, we updated the model for *Shewanella oneidensis* MR-1 and constructed metabolic models for three other strains, namely *Shewanella sp.* MR-4, *Shewanella sp.* W3-18-1, and *Shewanella denitrificans* OS217 which span the genus based on the number of genes lost in comparison to MR-1. We also constructed a *Shewanella* core model that contains the genes shared by all 21 sequenced strains and a few non-conserved genes associated with essential reactions. Model comparisons between the five constructed models were done at two levels – for wildtype strains under different growth conditions and for knockout mutants under the same growth condition. In the first level, growth/no-growth phenotypes were predicted by the models on various carbon sources and electron acceptors. Cluster analysis of these results revealed that the MR-1 model is most similar to the W3-18-1 model, followed by the MR-4 and OS217 models when considering predicted growth phenotypes. However, a cluster analysis done based on metabolic gene content revealed that the MR-4 and W3-18-1 models are the most similar, with the MR-1 and OS217 models being more distinct from these latter two strains. As a second level of comparison, we identified differences in reaction and gene content which give rise to different functional predictions of single and double gene knockout mutants using Comparison of Networks by Gene Alignment (CONGA). Here, we showed how CONGA can be used to find biomass, metabolic, and genetic differences between models.

**Conclusions:**

We developed four strain-specific models and a general core model that can be used to do various *in silico* studies of *Shewanella* metabolism. The developed models provide a platform for a systematic investigation of *Shewanella* metabolism to aid researchers using *Shewanella* in various biotechnology applications.

## Background

*Shewanella* is a genus of facultatively anaerobic, Gram-negative aquatic bacteria found in diverse environments around the globe [1]. This ecological diversity is enabled by their highly adaptable metabolism for which they have a diverse respiratory system, capable of reducing up to 20 different organic and inorganic compounds [1,2]. In addition, their carbon usage is quite varied, mainly comprising two- and three-carbon fermentation products, amino acids, and sugars [1,3,4]. *Shewanella* are of particular interest today because of their possible use in bioremediation in which the organisms convert a wide variety of metals from a soluble to an insoluble form and thus prevent the spread of contamination [5]. For example, *Shewanella putrefaciens* has been shown to reduce soluble uranium-VI to insoluble uranium-IV [6]. *Shewanella* strains can also degrade halogenated organics, including polychlorinated biphenyls and possibly explosive nitramines [7]. *Shewanella* strains have also been metabolically engineered for chemical production [8].

Currently, there are over 20 sequenced strains of *Shewanella*. These genomic datasets provide insight into both the cumulative (or pan) and conserved (or core) capabilities of this species. Previous studies have used these sequenced genomes to study metabolic subsystems [3] and regulons [9] in *Shewanella* strains. Another recent study looked at the carbon, nitrogen, phosphorous and sulfur utilization capabilities of five different *Shewanella* strains and reconciled these with genomic data [10].

Metabolic modeling provides a way to integrate the wide variety of data available on the *Shewanella*, from both traditional microbiology and high-throughput “-omics” (summarized in [7]) experiments. In addition, genome-scale metabolic models provide a systematic way to assess the metabolic potential of an organism [11,12]. With genome sequences available for more than 20 *Shewanella* strains [2,13], models can be used to improve our understanding of metabolism in the *Shewanella* genus as a whole [2], which will allow model-based predictions of the behavior of unstudied strains.

The genome-scale metabolic model of *Shewanella oneidensis* MR-1 published in 2010 (referred to as *i*SO783) has been used as a platform to integrate and validate experimental data with computational predictions [11]. This model also provides a platform to develop other genome-scale metabolic models for other *Shewanella* strains. In this study, we have expanded the previous model for MR-1 based on updated genome annotations and compared model growth predictions to fitness measurements for transposon-tagged mutants [4]. We subsequently used the updated model (hereafter *i*MR1_799) together with genome comparisons (based on genome annotations) and experimental data to develop the genome-scale metabolic models for three other *Shewanella* strains, *Shewanella* sp. MR-4 (hereafter *i*MR4_812), *Shewanella* sp*.* W3-18-1 (hereafter *i*W3181_789), and *Shewanella denitrificans* OS217 (hereafter *i*OS217_672). In addition, we developed a *Shewanella* core (hereafter Core) model using genome annotations of 21 sequenced *Shewanella* strains that would represent the conserved metabolic functionalities of all *Shewanella* strains. Furthermore, we used the developed models to predict and compare the metabolic capabilities of *Shewanella* strains in utilizing various carbon and electron acceptor sources. We also used a previously developed computational algorithm, Comparison of Networks by Gene Alignment (CONGA) [14] to identify functional differences between the developed metabolic networks, which helps reveal unique metabolic and genetic differences in each *Shewanella* strain.

## Methods

### Strains and media

*Shewanella oneidensis* MR-1 was obtained from Grigoriy Pinchuk (Pacific Northwest National Laboratory), *Shewanella* sp. MR-4 and *Shewanella* sp. W3-18-1 were obtained from Daad Saffarini (University of Wisconsin-Milwaukee). *Shewanella denitrificans* OS217 was obtained from the American Type Culture Collection (Manassas, VA). Strains were cultivated at 30°C in Luria Broth (LB) (*S. oneidensis* MR-1, MR-4, and W3-18-1), half-strength Difco Marine Broth (*S. denitrificans* OS217) or modified M1 medium [11]. For growth phenotype experiments modified M1 medium was supplemented with various carbon sources at 40 mM concentration.

### Growth phenotype experiments

For growth rate experiments, strains were precultured overnight in 2 mL LB or Marine Broth at 30°C with continuous shaking. Cells were then transferred into 2 mL of modified M1 supplemented with the carbon source of interest at 20 mM concentration and 20 mM D, L-lactate (MR-1, MR-4, and W3-18-1) or 20 mM maltose (OS217) using a 1:100 dilution from the overnight LB or Marine Broth culture and grown for 24 hours (MR-1, MR-4, and W3-18-1) or 48 hours (OS217) at 30°C. The cells were then harvested by centrifugation at 5000 rpm and resuspended in modified M1 medium containing no carbon source to an OD600 of 1–1.5. 5 μL of the cell suspension was then added to 95 μL of modified M1 medium supplemented with the carbon source of interest on a 96-well plate.

Growth/no-growth phenotype tests were performed using the same procedure, with the second preculture containing only lactate or maltose at 40 mM concentration. After harvesting, the cells were washed with 1 mL of modified M1 medium to remove any residual carbon source, centrifuged again, and resuspended. Growth curve experiments were conducted in a Tecan Infinite M200 plate reader (Tecan Group Ltd., Switzerland). Cultures were grown at 30°C in 96 well plates with OD600 readings taken every 15 minutes. Growth rates (1/h) were calculated from the linear fit of ln(OD) versus time, where the slope corresponds to the growth rate. Biomass yields (OD/mmol) were calculated by subtracting the starting OD from the stationary phase OD and dividing by the starting concentration of the carbon substrate. Results are provided in Additional file 1: Figure S1.

### Identification of orthologs

A table with the draft ortholog predictions among the 21 sequenced *Shewanella* strains was constructed using INPARANOID as previously described [15]. This table was subjected to extensive manual curation to improve the prediction of ortholog group membership. Gene synteny among the *Shewanella* strains made it possible to readily identify orthologous groups that had missing members or that contained extra or erroneously grouped members (typically groups containing laterally acquired genes). Missing group members were identified by tBLASTN analysis (genes missing in gene models) or BLASTP analysis (proteins missing due to the presence of paralogs in one of the genomes resulting in improper resolution of groups or due to sequence similarity lower than the defined cut-off). Insertion elements were mapped in each of the genomes as described for *S. oneidensis* MR-1 [16] to assist in identification of gene fragments; these “pseudogenes” (denoted by “^” in the ortholog table) were also added (using BLASTP of protein fragments to identify orthologs) to the ortholog table. Comparison of domain content and predicted subcellular location among group members were then used to refine group membership as previously described [17]. The ortholog table is presented in Additional file 2: Table S1.

### Model reconstruction of the five *Shewanella* models including the Core

The *Shewanella* models *i*MR4_812, *i*W3181_789, and *i*OS217_672 developed in this study were constructed manually using the *i*MR1_799 as a template, and the gene-protein-reaction (GPR) associations in each new model were constructed based on the ortholog table. Reactions in the *i*MR1_799 metabolic model were copied to the metabolic models of these strains if the associated MR-1 genes had orthologs in the other strains or if no genes were associated to the reaction in *i*MR1_799. Genes that did not have orthologs in these strains and their associated reactions were removed from the base *i*MR1_799 model to obtain draft models that were specific to *i*MR4_812, *i*W3181_789, and *i*OS217_672. Flux balance analysis (FBA) [18] was then used to predict growth of these draft models upon removing reactions from the base model. If the deleted reaction prevented growth in the model that conflicted with experimental data, genes (and associated reactions) with similar functions identified in the genome annotation were added to the constructed model. Furthermore, metabolic genes that are unique and specific to each strain and their associated reactions were added into the models. The biomass and lipopolysaccharide (LPS) reactions were updated if necessary to reflect the physiology of the organism. For example, the LPS reaction for *i*W3181_789, *i*OS217_672, and Core was modified because unlike *i*MR1_799, they are not capable of producing UDP-N-acetyl-D-galactosamine. The LPS reaction for *i*MR4_812 was updated based on the structure determined experimentally by Vinogradov et al. [19].

The Core model was constructed based on the genes that were conserved across all 21 sequenced *Shewanella* strains. Non-GPR reactions were added to the model only if they were predicted to be essential for aerobic growth on pyruvate, resulting in a smaller number of reactions without GPR associations (see Table 1). Furthermore, there are four essential reactions in the Core model with GPRs that include genes that are not conserved across all 21 *Shewanella* strains. For example, the first reaction glutamate-5-semialdehyde dehydrogenase (G5SD) converts L-glutamate 5-phosphate (glu5p) to L-glutamate 5-semialdehyde (glu5sa). Every *Shewanella* strain studied has a ProA enzyme that catalyzes the G5SD reaction; however, there are two different orthologs that encode the ProA enzyme, and some strains have only one of the two. Since the Core model represents the collective group and G5SD is an essential reaction (meaning it has to stay in the Core model), the two genes associated with ProA were encoded as isozymes in the Core model. The other three reactions are acetylornithine deacetylase (ACODA), inorganic diphosphatase (PPA), and a sulfate transport reaction (SO4t2).

**Table 1:** ***Shewanella *** **strain-specific metabolic model statistics comparison**

	***i*** **SO783**	***i*** **MR1_799**	***i*** **MR4_812**	***i*** **W3181_789**	***i*** **OS217_672**	**Core**
**Number of reactions**	870	933	986	918	865	673
**- Reactions w/ GPR**	729	758	794	749	696	591
**- Reactions w/o GPR**	46	60	65	60	65	21
**- Exchange reactions**	95	115	127	109	104	61
**Number of genes**	783	799	812	789	672	552
**Number of metabolites**	634	647	665	643	638	565
**Percent of reactions in Core**	N/A	72%	68%	73%	78%	100%
**Percent of genes in Core**	N/A	69%	68%	70%	82%	100%

The complete list of reactions, metabolites, and genes for all five *Shewanella* models can be found in Additional file 2: Tables S2-S4. All five models are also available in SBML format in Additional file 3. A list of compounds included in the different biomass equations is provided in Additional file 1: Figure S2.

### Evaluation of mutant fitness data

The mutant fitness data set from Deutschbauer et al. [4] contains fitness scores for 3,355 knockout mutants for each of the 195 pool fitness experiments (for a total of over 650,000 fitness scores). A fitness score of −3.5 was used as a cutoff to distinguish mutants that grew or did not grow. This cutoff results in a similar percentage of viable mutants (out of 3,355) which were unable to grow in different conditions (~3% across all 195 experiments, Additional file 1: Figure S3) as found in a previous study with *Escherichia coli* (~3% of 3,888 mutants) [20]. In addition, Deutschbauer et al. individually tested 48 mutants, and found that those with a fitness score less than −3.5 had growth rates 60% lower than the wild type (4 out of 6) or had long lag phases (2 out of 6). Based on these results, we set the cutoff for growth based on fitness scores to be −3.5 (i.e., fitness scores less than −3.5 indicated no growth and fitness scores greater than −3.5 indicated growth). If there are multiple experiments for the same simulated condition (e.g., carbon source experiments with or without 1 mM calcium chloride is considered the same simulated condition), then the mutant will be assigned as growth if the majority of similar experiments have z-scores above the cutoff, or no growth if the majority of similar experiments have z-scores below the cutoff. However, if there are equal numbers of entries with z-scores above and below the cutoff, then the growth of the mutant is deemed undetermined. These undetermined cases were not considered when calculating the accuracy, false positive, and false negative prediction rates of the models.

Only experiments from the “carbon source”, “nitrogen source”, “carbon and nitrogen source”, and the “anaerobic” groups where considered. Experiments from the remaining groups were not considered because they cannot be simulated using the models. Next, experiments that are labeled as stationary phase, saturated, or with added CAS amino acids were excluded from our analysis. In total, we were able to make 35,712 predictions for 558 of the 3,355 knockout mutants (other mutants involved genes that were not in the model) under 64 unique simulation conditions for the *i*MR1_799 model. The 64 simulation conditions can be mapped to 101 of the 195 fitness experiments (the number of fitness experiments is higher due to replicates and the way experiments were simulated by the model, as described above). Additional file 2: Table S5 contains a list of the 64 different simulation conditions and what sets of experimental data were used to compare predictions to.

### Model-predicted growth phenotypes on different carbon sources and electron acceptors

**Figure 1:**
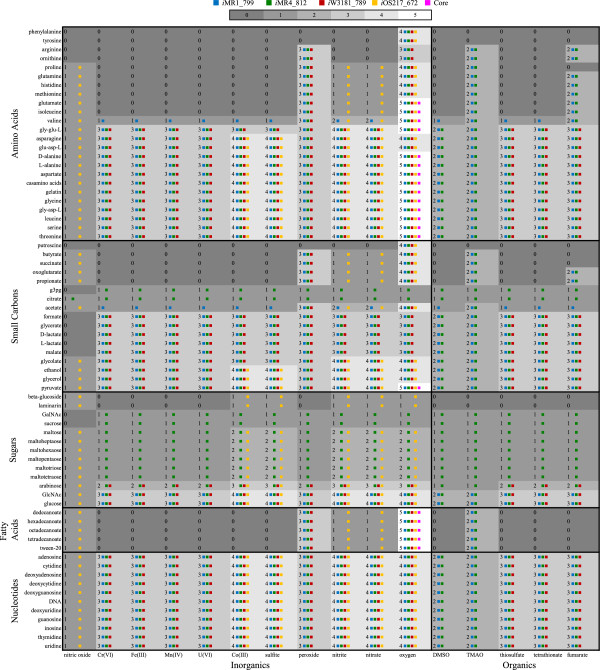
**Predicted growth phenotypes of *i*MR1_799, *i*MR4_812, *i*W3181_789, *i*OS217_672, and Core metabolic models.** The number in each cell represents the number of strains predicted to be able to grow on the corresponding pair of carbon source (rows) and electron acceptor (columns) and the small colored squares to the right of each number indicate the growing strain(s). g3pg, glycerophosphoglycerol; GalNAc, N-acetylgalactosamine; GlcNAc, N-acetylglucosamine; Cr(VI), chromate; Fe(III), iron(III); Mn(IV), manganese(IV) oxide; U(VI), uranyl; Co(III), cobalt(III); DMSO, dimethyl sulfoxide; TMAO, trimethylamine N-oxide.

FBA [18] was used to predict growth by different *Shewanella* models (*i*MR1_799, *i*MR4_812, *i*W3181_789, *i*OS217_672, and Core) on different carbon sources and electron acceptors (see Figure 1 for complete listing). Briefly, for each carbon source and electron acceptor tested, flux through the biomass reaction was maximized. The exchange fluxes for a particular carbon source and electron acceptor were constrained to have lower bounds of −10 mmol/g AFDW/h (where g AFDW is grams ash free dry weight). In all simulations, the lower bounds on the exchange fluxes for phosphate, sulfate, water, proton, and ammonia (if ammonia was the nitrogen source) were set to −1000 mmol/g AFDW/h. The upper bound on all exchange fluxes was 1000 mmol/g AFDW/h so that any extracellular compound could be secreted. Growth- and non-growth-associated ATP requirements were set to zero for all model simulations since these values are unknown for the other strains. The sets of carbon sources and electron acceptors that were tested in the simulations were the union sets of carbon sources and electron acceptors from the five metabolic models. All models used the same exchange flux constraints.

### Cluster analysis

A hierarchical clustering approach was used to gain an understanding of how the four *Shewanella* models relate to each other under different conditions. A heat map was generated and used to create a dendrogram using the “clustergram” function in MATLAB. Clustergram takes in an input matrix of interest and generates a dendrogram based on a specified distance metric. The default ‘euclidean’ distance metric was used in this analysis. The height of each branch represents the distance between the two data points that are connected. The heat maps were generated using binary matrices indicating whether a metabolic gene is present (1) or absent (0) in a particular model or whether the model was predicted to grow (1) or not (0) under a given growth condition.

### Network comparison using CONGA

CONGA, or Comparison of Networks by Gene Alignment [14], is a constraint-based method that can be used to identify how differences in reaction content or GPR associations give rise to differences in growth predictions between models by comparing reconstructed networks aligned at the gene level. The method identifies gene deletion strategies that lead to different optimal flux distributions in a pair of networks. Specifically, CONGA identifies a set of orthologous genes to delete such that the flux difference in a reaction of interest (e.g., growth rate) between the two models is maximized. The set of orthologs deleted by CONGA are referred to as a gene deletion set. In this paper, we were interested in the gene deletion sets that when knocked out from both organisms would leave only one capable of growth, and so we chose to maximize the difference in flux through the biomass reaction. Please refer to the original paper on CONGA for additional algorithm details [14].

## Results and discussion

### Development of five *Shewanella* models including the Core

**Figure 2:**
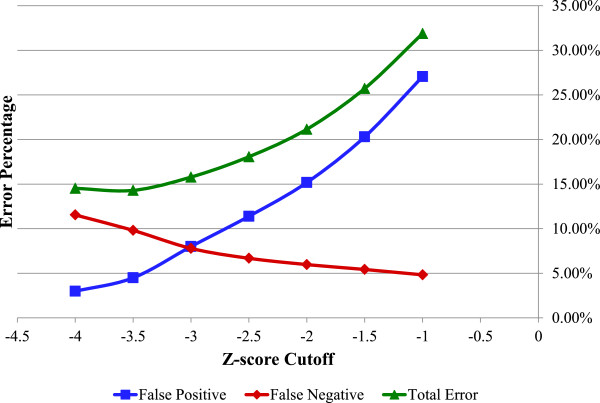
**Sensitivity analysis of *i*MR1_799 model predictions to the z-score cutoffs on experimental data.** Shown here are the false positive (blue squares), false negative (red diamonds), and overall prediction errors (green triangles) by the *i*MR1_799 model given different z-score cutoffs for the 3,355 knockout mutants under 64 conditions. A false positive result indicates that the model predicted growth while experimental data shows no growth. A false negative result is the opposite where the model predicted no growth while experimental data shows growth.

Compared to the original model for MR-1 (*i*SO783) [11] our updated model (*i*MR1_799) contains additional reactions involved in alternate carbon metabolism (5), cell envelope biosynthesis (1), energy metabolism (3), glycolysis/gluconeogenesis (1), methionine metabolism (1), nucleotide salvage pathways (5), valine, leucine, and isoleucine metabolism (8), and additional transport reactions (19). To evaluate the performance of the original and updated MR-1 models, we compared model predictions to a recent study reporting wildtype and mutant phenotypes [4]. Of the 195 experiments evaluated in the original study, we were able to map 101 experiments to 64 different simulatable conditions that *i*MR1_799 predicted could support growth (see Methods for details). The *i*SO783 model could only predict growth for the wildtype strain in 40 of these 64 conditions, indicating that 24 conditions are incorrectly predicted by the original model. When evaluating the growth phenotypes for the subset of mutants (546) and conditions (40) that both models could make predictions for, we found the *i*SO783 model had an overall accuracy of 85.11% and the *i*MR1_799 model had an overall accuracy of 85.34%. This was done assuming that mutants with a fitness score less than −3.5 did not grow (see Methods for details). We next evaluated the sensitivity of the *i*MR1_799 model’s accuracy to the fitness cutoff used to indicate growth or no growth phenotypes (see Figure 2) using all mutants (558) and conditions (64) that could be simulated by *i*MR1_799. A summary of the 55 mutants that the *i*MR1_799 failed to predict correctly in at least half of the conditions is provided in Additional file 2: Table S6 along with possible reasons for model-data discrepancies.

The updated *i*MR1_799 was used as a starting point to generate models for three additional strains. Several alternative carbon metabolism pathways were added to the *Shewanella* models based on literature data [3] and our experimental observations. For example, three *Shewanella* strains (MR-4, W3-18-1, and OS217) were shown to be able to use arabinose as a carbon source under aerobic conditions (see Additional file 2: Table S7). Although only *i*MR4_812 and *i*W3181_789 have the genes associated with arabinose catabolism, the reactions were also added to *i*OS217_672 without GPR associations. This accounts for why the number of reactions without GPR across the four models is different even though they all carried over the non-GPR reactions from *i*MR1_799 (see Table 1).

**Figure 3:**
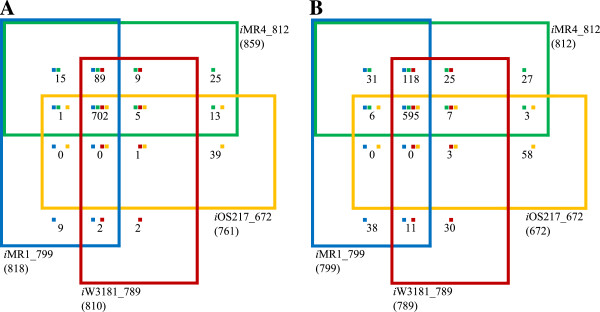
**Four-way Venn diagrams for four of the reconstructed metabolic models.** Four-way Venn diagrams for the number of reactions **(A)** and genes **(B)** in *i*MR1_799 (blue), *i*MR4_812 (green), *i*W3181_789 (red), and *i*OS217_672 (orange) metabolic models. The number in parenthesis next to the model name outside of each rectangular box indicates the total number of reactions (panel A) or genes (panel B) in that model. Note that total number of reactions here excludes exchange reactions to better represent metabolic reactions in the models. The numbers within the boxes indicate the total number of reactions (panel A) or genes (panel B) that are shared among the overlapping models. The small colored squares above these numbers indicate the models that are overlapping.

As shown in Table 1, the total number of reactions in the *i*OS217_672 model is much lower than the other three models because *i*OS217_672 has the most number of deleted reactions from the *i*MR1_799 model (see Table 2). To better compare content between the four models, we generated 4-way Venn diagrams to show the number of reactions and genes that are shared or are unique between each model. All four models share 702 reactions (including both GPR and non-GPR reactions, excluding exchange reactions) (Figure 3A). Furthermore, *i*MR1_799, *i*MR4_812, and *i*W3181_789 share 89 reactions that are absent in *i*OS217_672. It should be noted that *i*OS217_672 appears to be the most distinct among these 4 models since it has the most number of unique reactions and shares the least number of reactions with the other models. A similar pattern was found with the number of orthologs shared among the 4 models (Figure 3B). All four models share 595 metabolic genes. Again, *i*OS217_672 appears to be the most distinct strain based on the high number of unique genes and the low number of shared genes with the other models.

**Table 2:** **Number of reactions and genes lost and gained in comparison to *i*** **MR1_799**

	***i*** **MR4_812**	***i*** **W3181_789**	***i*** **OS217_672**	**Core**
**Reactions lost**	11	33	139	262
**Reactions gained**	64	18	71	2^a^
**Genes lost**	49	75	198	251
**Genes gained**	62	65	71	4^b^

^a^The two unique reactions in the Core model with not in *i*MR1_799 are the lipopolysaccharide synthesis reaction and the biomass reaction.

^b^The four genes in the Core model that are not in *i*MR1_799 are defined as isozymes for the glutamate-5-semialdehyde dehydrogenase (G5SD), acetylornithine deacetylase (ACODA), inorganic diphosphatase (PPA), and sulfate transport (SO4t2) reactions since some *Shewanella* strains have different non-orthologous genes that catalyze these reactions.

The Core model was built to provide a representation of the *Shewanella* genus by only including the reactions and genes that are conserved across the 21 sequenced strains and a few additional essential reactions (see Methods for details). Only 21 essential reactions without GPR associations were kept in the Core model to better represent a minimal model of the *Shewanella* genus, of which 17 of these 21 reactions do not have GPR associations in any of the models. The remaining four non-GPR core reactions point towards gaps in our knowledge for some of the 21 *Shewanella* strains. The 673 reactions and 552 genes in the Core model turned out to represent an average of 73% and 72% of the reactions and genes of the 4 *Shewanella* models developed here, respectively (see Table 1 for a detailed percentage breakdown for each model). This means that on average, the remaining 27% of the reactions and 28% of the genes are responsible for extra metabolic capabilities specific to each strain. This is comparable to the percentage of genes in the core *E. coli* model developed by Monk et al. [21] out of 55 *E. coli* models (965 core genes/1305 average genes per model = 74%).

By comparing the wildtype version of each of these five models, we are able to identify how each model and thus strain is physiologically different from the entire *Shewanella* genus. In addition, by comparing knockout predictions across the five models, we are able to identify deeper genetic and metabolic differences for each strain. We explore these two levels of comparison in the following sections.

### Model-predicted growth phenotypes on different carbon sources and electron acceptors

The reconstructed metabolic models of different *Shewanella* strains, *i*MR1_799, *i*MR4_812, *i*W3181_789, *i*OS217_672, and the Core model were used to predict growth capabilities of each strain on different carbon sources and electron acceptors. A total of 70 carbon sources and 16 electron acceptors were used in the FBA simulations (see Figure 1 for listing of compounds tested). Among these 1,120 pairs of carbon sources and electron acceptors, there were 47 pairs that have been verified experimentally to support growth of MR-1 [4]. Comparing only the aerobic cases, we found that our models correctly predicted 35 out of 40 carbon sources for *i*MR1_799 (88%), 31/36 for *i*MR4_812 (86%), and 25/36 for *i*W3181_789 (69%) when compared against experimental data [3,4,10,11], including some generated in this study (Additional file 2: Table S7). Note that whenever there was a conflict between the experimental results, we assumed that the high-throughput methods were less accurate. Some of the inaccurately predicted cases were for compounds that were shown experimentally to serve as sole nitrogen sources (3 separate cases for each model). Based on the nitrogen source results, the pathways to metabolize those compounds should be in the models, which allow the models to also predict their utilization as carbon sources. However, the fact that those compounds were shown to be used as sole nitrogen sources and not sole carbon sources suggests that there might be regulatory limitations within the strains that prevent them from using those compounds as sole carbon sources.

For a particular pair of carbon source and electron acceptor, growth phenotypes were qualitatively classified as growth if the model predicted growth rate is positive and no growth if the predicted growth rate is zero. The number on the heat map indicates the number of *Shewanella* strains that were predicted to grow on the corresponding carbon source and electron acceptor (see Figure 1). As expected, under aerobic conditions (with oxygen as electron acceptor) most models (4 or 5 models) were predicted to grow with a variety of carbon sources (amino acids, small carbon compounds, nucleotides, and fatty acids). There were at most three models that were predicted to be able to grow using organic electron acceptors while most models were predicted to use inorganic electron acceptors (such as cobalt, nitrite, or nitrate). Fumarate was predicted to be the organic electron acceptor that can be used in combination with a majority of carbon sources to enable *in silico* growth. While the number of models that were predicted to grow on different electron acceptors varied greatly with different amino acids and small carbon compounds, it was quite consistent across nucleotides, sugars, and fatty acids. This indicated that the enzymes or pathways that utilize these latter carbon sources were the same in each strain, or the strain had enzymes that convert one carbon source to another.

### Cluster analysis of the reconstructed models

**Figure 4:**
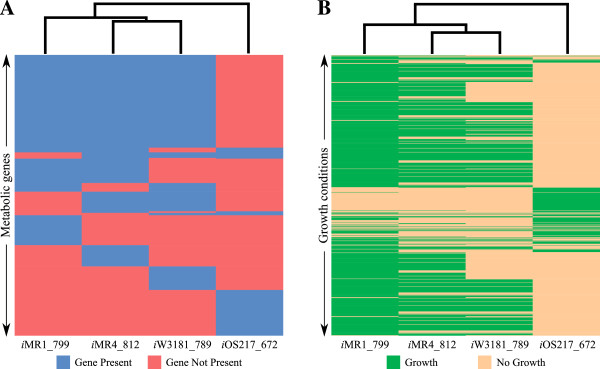
**Cluster analysis of the four *Shewanella *models based on different criteria.***In silico Shewanella* cluster analysis including a dendrogram and a heat map for *i*MR1_799, *i*MR4_812, *i*W3181_789, and *i*OS217_672 based on metabolic genes **(A)**, and predicted growth phenotypes **(B)**. Each leaf spaced evenly along the horizontal axis of the dendrogram and each column in the heat map represents each *Shewanella* model. The vertical axis of the dendrogram indicates a distance measure. The height of each branch represents the distance between the two clusters. The rows in the heat map represent different growth conditions (i.e., carbon source and electron acceptor). The heat maps indicate whether the metabolic gene is present in each strain (panel A) and whether the strain is predicted to grow in the given growth condition (panel B). Note that genes that are present in all four strains and growth conditions where all four strains had the same predictions (growth or no growth) were omitted from this figure for clarity. This omission does not affect the clustering results.

To evaluate how each model relates to the others based on the presented results so far, heat maps and dendrograms were created for the four models based on metabolic gene content or phenotype predictions (see Methods). First, a heat map and dendrogram was generated based on the presence or absence of metabolic genes in the metabolic models (Figure 4A). From this analysis, *i*MR4_812 and *i*W3181_789 were found to be the most similar, followed by *i*MR1_799 on the left and *i*OS217_672 on the right of the first pair, indicating that *i*MR1_799 and *i*OS217_672 are the most different. Additional analysis revealed that the orders between *i*MR1_799, *i*MR4_812, and *i*W3181_789 can switch with just a few changes in the genes considered due to the small differences in the dendrogram branch heights. Next, a heat map and dendrogram was generated based on predicted growth phenotypes on the different carbon sources and electron acceptors (Figure 4B). This time, while *i*OS217_672 is still the most distinct strain, *i*MR1_799 is more similar to *i*W3181_789 than *i*MR4_812 in the dendrogram (this is due to the predicted ability of only *i*MR4_812 to use maltodextrins). This result suggests that while *i*MR4_812 and *i*W3181_789 have highly similar metabolic gene content, the genes deleted from *i*MR1_799 and those added to *i*MR4_812 and *i*W3181_789 resulted in *i*MR1_799 and *i*W3181_789 being more metabolically similar from a network perspective.

### Multi-model network comparison using CONGA

CONGA is a constraint-based method that can be used to identify functional differences between two models by comparing reconstructed networks aligned at the gene level. We used CONGA to identify orthologous genes to delete such that only one of the two organisms being compared is capable of growth. In this work, we identified three reasons that explain the functional differences found by CONGA: metabolic (e.g., a unique pathway), genetic (e.g., a unique isozyme), and biomass differences (Table 3, see Additional file 1: Figure S4 and text for a detailed description along with illustrated examples of CONGA results).

**Table 3:** Types of CONGA results

**Types**	**Definition**	**Examples from this study**
Biomass	Two models being compared have different biomass reactions which cause differences in gene essentiality	Deletion of genes associated with reactions that produce putrescine or spermidine is lethal in all models except the Core because these compounds are not in the Core biomass reaction
Metabolic	One organism possesses an alternative pathway for an essential reaction	Deletion of genes associated with prephenate dehydrogenase is lethal only in the Core because it does not have an alternative pathway of producing L-tyrosine that the other models have
Genetic	Difference in the GPR associations between models for essential reactions	Deletion of AsnB (SO2767) is lethal in all models except for *i*MR1_799 because MR-1 has another isozyme for asparagine synthase, WbpQ (SO3175)

**Figure 5:**
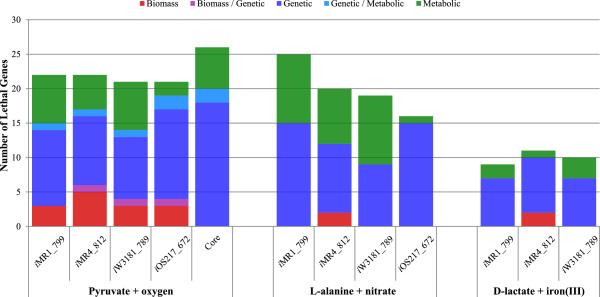
**CONGA results summary for single deletion case under all three conditions.** Shown here are the number of unique lethal genes identified by CONGA for each model that are not lethal in at least one other model. The numbers are split into the three types of differences; biomass, metabolic, and genetic difference. Cases where a lethal gene is caused by different types of differences depending on the models being compared are shown.

A summary of the CONGA results for the single gene deletion cases under three different media conditions can be found in Figure 5. To simplify the presentation, we combined all the pair-wise CONGA results to show the total number of unique lethal genes identified by CONGA for each model (i.e., deletion of the ortholog is lethal in one model but not in at least one other model). For example, for the aerobic pyruvate condition, we can see that there are 22 genes that are only essential in *i*MR1_799 but not in at least one of the other 4 models (Figure 5). There are cases where the deletion of one gene may be due to both genetic and metabolic differences depending on which models it is being compared to. For example, the deletion of MR4_2874 is lethal in *i*MR4_812 but the deletion of the orthologous *i*MR1_799 gene is not because *i*MR1_799 has two isozymes for the same reaction, thus, a genetic difference. On the other hand, the deletion of MR4_2874 is picked up by CONGA as being lethal in *i*MR4_812 and non-lethal in the Core model due to a biomass difference. Analysis for the double deletion cases can be found in Additional file 1: Figure S5. Original pairwise growth/no-growth CONGA results can be reconstructed using Additional file 2: Tables S8 and S9.

Based on this analysis, we can clearly see that under the aerobic condition, the Core model is the least robust since the Core model has the least number of genes and reactions. Thus, the Core model has fewer isozymes (with the exception of G5SD, ACODA, PPA, and a sulfate transporter, SO4t2) and alternative pathways to compensate for deleted genes. *i*MR1_799, *i*MR4_812, *i*W3181_789, and *i*OS217_672 have comparable robustness under this condition. However, under the L-alanine plus nitrate condition, *i*OS217_672 appears to be the most robust model. The robustness of *i*MR1_799 and *i*MR4_812 is actually comparable in this latter case but *i*MR1_799 appears to have a higher number of lethal genes because some of the reactions involved have many subunits. Thus, the same reaction is picked up by CONGA multiple times because deletion of any of the subunits produced the same results. For example, four of the genetic difference entries were all due to *i*MR1_799 not having a 4-subunit isozyme for the nitrate reductase reactions NTR4 and NTR5 which is present in both *i*MR4_812 and *i*W3181_789. Therefore, deletion of each of the four subunits results in a gene deletion set where *i*MR1_799 is lethal but not *i*MR4_812 or *i*W3181_789 under L-alanine plus nitrate conditions. Finally, *i*MR1_799, *i*MR4_812, and *i*W3181_789 have relatively similar robustness under D-lactate plus iron (III) conditions. The total number of lethal genes is lower in this condition because only three models were analyzed. The *i*OS217_672 and Core models were excluded from the last condition because they are not capable of growth under that condition.

Furthermore, we mapped the reactions associated with the genes in the gene deletion sets to their respective metabolic subsystems to see if a specific subsystem was responsible for most of the differences identified by CONGA for the single deletion case. Of all subsystems, the Energy Metabolism subsystem stood out with the most number of reactions associated with orthologs deleted by CONGA (14 reactions) and they all appear only under L-alanine plus nitrate conditions. Further investigation shows that all 14 reactions involve the gene associated with the membrane-anchored tetraheme cytochrome c, CymA. Deletion of CymA is lethal in *i*MR1_799 and not in *i*MR4_812 and *i*W3181_789 because the latter two models have alternative enzymes that do not require CymA for nitrate reductase activity.

## Conclusion

In this study, we have shown an extensive comparison of four different *Shewanella* strains, MR-1, MR-4, W3-18-1, and OS217 in terms of growth phenotypes using integrated approaches of genome comparisons, experiments, and computational models. Based on conservation of metabolic gene content, we expected that *i*MR4_812 and *i*W3181_789 to be the most similar, with *i*MR1_799 and *i*OS217_672 being the most different. However, our results revealed that based on predicted growth phenotypes MR-1 is most similar to W3-18-1, followed by MR-4 and OS217 in terms of metabolic capabilities in using various carbon and electron acceptor sources. This suggested that genetic similarity does not always coincide with metabolic phenotype similarity. Some conflicts between our models’ predictions and experimental data suggest there may be regulatory effects affecting cellular phenotypes that are not accounted by our models. Regulons for 82 transcription factors have been predicted based on comparative genomic analysis of 16 *Shewanella* strains and they also appear to highly vary across the different strains [9]. Together the metabolic and regulatory networks help shape the diversity of this species.

The Core model reflects the conserved metabolic capabilities of all sequenced strains. Based on analysis of the Core model, it appears that all strains appear to be capable of using pyruvate, amino acids (10 of 20), and fatty acids as sole carbon sources. Interestingly, only oxygen was predicted to be used as an electron acceptor by the Core model, indicating that while many strains can use other electron acceptors, the respiratory pathways and enzymes involved are not necessarily conserved.

We also used computational approaches to further investigate the metabolic and genetic differences among these strains under several common growth conditions. Our computational analyses provided more insights into the metabolic capabilities of each *Shewanella* strain of interest. Each genome-scale metabolic model developed in this work will serve as a platform to integrate experimental data with computational approaches for each *Shewanella* strain, which would be helpful to researchers who study *Shewanella* for scientific discovery, bioremediation, or metabolic engineering applications. In addition, the *Shewanella* core model will serve as a base model to develop genome-scale models for other *Shewanella* strains by simply adding metabolic functionalities that are unique to those individual strains.

## Competing interests

The authors declare that they have no competing interests.

## Authors’ contributions

WO, TV, and JL constructed the five genome-scale metabolic network models. KL ran the experiments. MS and MR analyzed the genomes and provided the ortholog table. WO and TV did the model simulations. WO, TV, KL, and JR analyzed and interpreted the modeling and experimental data. JR conceived of the study. WO, TV, KL, and JR wrote the manuscript. WO generated all five SBML model files. All authors read and approved the final manuscript.

## Supplementary Material

Additional file 1 **Supplementary material including supplementary text and figures.** This file contains additional discussion on the growth experiments for MR-1 and W3-18-1, biomass composition, evaluation of mutant fitness data, and CONGA. Click here for file

Additional file 2 **Supplementary tables.** This file contains additional tabular data. Click here for file

Additional file 3 **XML files for the five *Shewanella *models in SBML format.** This file contains the XML files for the five models constructed in this work. Click here for file
