# Supplementary material for: Comparisons of Shewanella strains based on genome annotations, modeling, and experiments
Source: BMC Syst Biol. 2014 Mar 12;8:31. doi: 10.1186/1752-0509-8-31 (PMC4007644; doi:10.1186/1752-0509-8-31)
Supplement: Additional file 1 — Supplementary material including supplementary text and figures. This file contains additional discussion on the growth experiments for MR-1 and W3-18-1, biomass composition, evaluation of mutant fitness data, and CONGA. [file 1752-0509-8-31-S1.pdf]

Supplementary Materials

Experimental growth comparisons between *S. oneidensis* MR-1 and *Shewanella* sp. W3-18-1

Given the predicted similarities in qualitative growth phenotypes between *S. oneidensis* MR-1 and *Shewanella* sp. W3-18-1 we conducted batch growth experiments to investigate possible quantitative differences in growth phenotypes. The growth rates and biomass yields were measured for both strains under aerobic conditions with different starting concentrations of carbon sources (Figure S1). In particular, W3-18-1 appeared to grow significantly faster than MR-1 on pyruvate, regardless of the substrate's concentrations (Figure S1A). In contrast, at lower substrate concentrations (e.g., 10 mM), MR-1 appeared to grow faster on L-lactate and D,L-lactate, while W3-18-1 grew faster at higher substrate concentrations (40 mM). For most conditions tested (except 10 mM D,L-lacate) MR-1 had lower biomass yields compared to W3-18-1 (Figure S1B) even though the models predict higher biomass yields for MR-1 than W3-18-1. The prediction differences are mainly due to differences in the aerobic respiratory enzymes between the two strains, where the *i*MR1_799 model has a maximum P/O ratio (ATP produced per electron pair transferred to oxygen via the electron transport chain) of 2.5 and the *i*W3181_789 model has a maximum P/O ratio of 1.5. The higher observed biomass yields for W3-18-1 indicates that the strain has potentially lower ATP requirements, lower futile cycle activity, and/or uses more energetically efficient pathways than MR-1. These observations are not predicted by our metabolic models since FBA predicts fluxes needed to achieve maximal biomass yields.

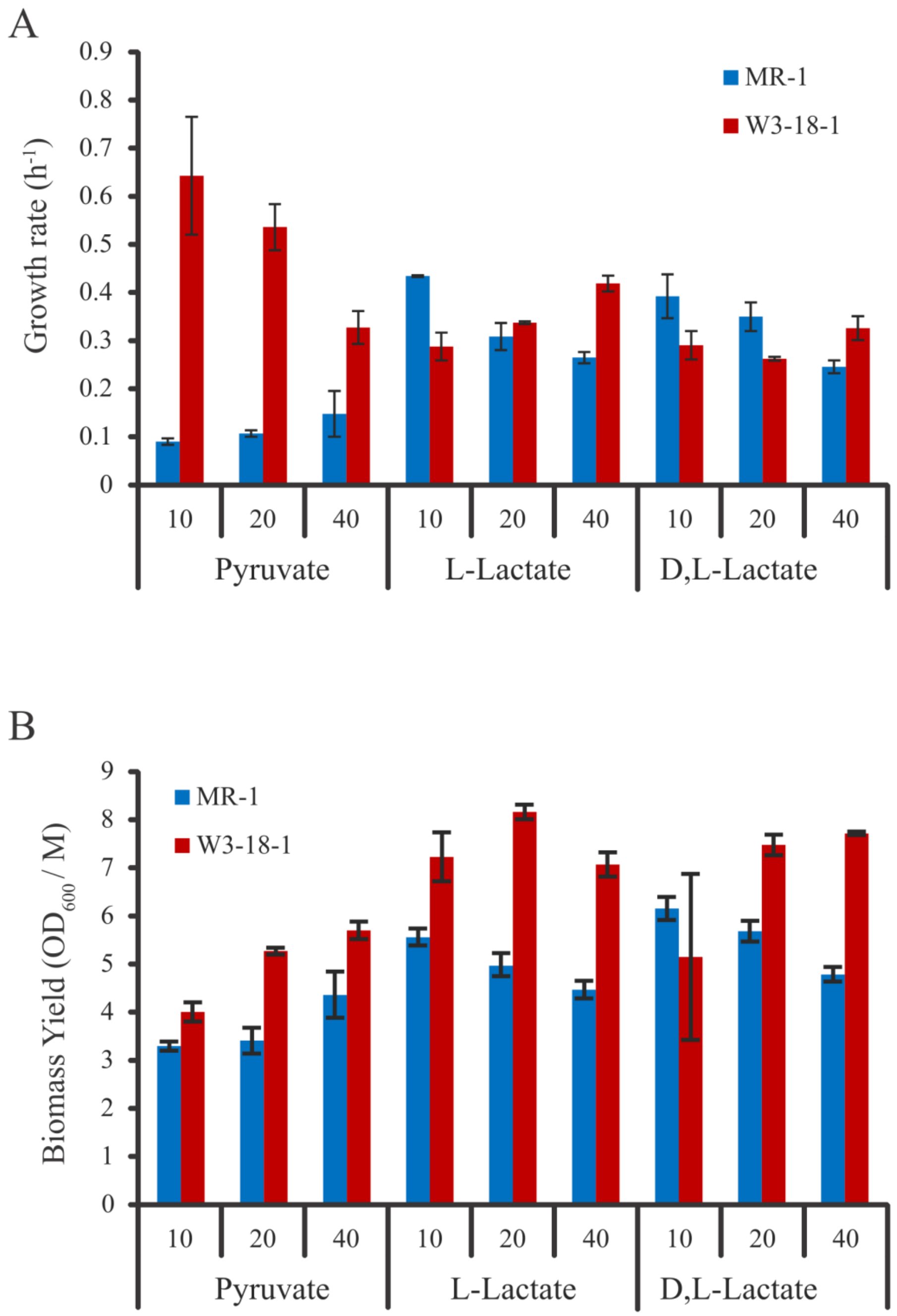

Figure S1. Growth rate comparison of *Shewanella oneidensis* MR-1 and *Shewanella* sp. W3-18-1. (A) Growth rates (h^{-1}) and (B) biomass yields (OD_{600}/M) of *Shewanella oneidensis* MR-1 (blue bars) and *Shewanella* sp. W3-18-1 (red bars) were calculated from triple replicates of growth experiments for strains grown aerobically in pyruvate, L-lactate, and D,L-lactate with three different initial concentrations of carbon sources.

Biomass composition and lipopolysaccharide reactions

The biomass composition for MR-1 was measured experimentally and detailed in Pinchuk et al. [1]. However, when building the model for MR-4, we found that it was missing the gene that produces the WbpP (SO3189) enzyme involved in lipopolysaccharide (LPS) synthesis. In fact, all the other 3 strains studied were missing this enzyme, which leads to the inability to produce UDP-N-acetyl-D-galactosamine (uacgala). Therefore, the reaction associated with LPS synthesis was modified based on the works by Vinogradov et al. [2] who used NMR, mass spectrometry, and chemical methods to determine the rough type LPS isolated from MR-4 (see Table S2 in Additional file 2). The LPS synthesis reaction for *i*MR4_812 now contains UDPgalacturonate (udpgalur). Having said that, W3-18-1 and OS217 both could not produce uacgala and udpgalur, and so have a simplified LPS synthesis reaction that was also used to represent the LPS synthesis reaction for the Core model. Finally, besides excluding ptrc and spmd from the Core biomass reaction, acyl-glycerophosphoethanolamine (agpe), acyl-glycerophosphoglycerol (agpe), and glycogen were also excluded from both *i*OS217_672 and the Core model because *i*OS217_672 is missing the genes associated with the reactions that produce them (see Figure S2 in below). Also, due to this change, the coefficients for the remaining biomass components for *i*OS217_672 were rescaled such that they add up to 1 g ash-free dry weight of biomass.

Compound	Abbreviation	*i*MR1_799	*i*MR4_812	*i*W3181_789	*i*OS217_672	Core
1,2-Diacylglycerol	12dgr	Present	Present	Present	Present	Present
1,2-Diacyl-sn-glycerol 3-phosphate	12dag3p	Present	Present	Present	Present	Present
DNA	dna_son	Present	Present	Present	Present	Present
Lipopolysaccharide (MR1)	lps_so	Present	Absent	Absent	Absent	Absent
Lipopolysaccharide (MR4)	lps_mr4	Absent	Present	Absent	Absent	Absent
Lipopolysaccharide (Core)	lps_core	Absent	Absent	Present	Present	Present
Peptidoglycan	peptx_e	Present	Present	Present	Present	Present
Phosphatidylethanolamine	pe	Present	Present	Present	Present	Present
Phosphatidylglycerol	pgly	Present	Present	Present	Present	Present
Protein	protein_son_aerobic	Present	Present	Present	Present	Present
RNA	rna_son	Present	Present	Present	Present	Present
5-Methyltetrahydrofolate	5mthf	Present	Present	Present	Present	Present
Acetyl-CoA	accoa	Present	Present	Present	Present	Present
AMP	amp	Present	Present	Present	Present	Present
Coenzyme A	coa	Present	Present	Present	Present	Present
FAD	fad	Present	Present	Present	Present	Present
NAD	nad	Present	Present	Present	Present	Present
NADH	nadh	Present	Present	Present	Present	Present
NADP	nadp	Present	Present	Present	Present	Present
NADPH	nadph	Present	Present	Present	Present	Present
Succinyl-CoA	succoa	Present	Present	Present	Present	Present
UDPglucose	udpg	Present	Present	Present	Present	Present
Putrescine	ptrc	Present	Present	Present	Present	Absent
Spermidine	spmd	Present	Present	Present	Present	Absent
Acyl-glycerophosphoethanolamine	agpe	Present	Present	Present	Absent	Absent
Acyl-glycerophosphoglycerol	agpg	Present	Present	Present	Absent	Absent
Glycogen	glycogen	Present	Present	Present	Absent	Absent

Present Absent

Figure S2. Biomass composition for each model. Differences in the biomass reaction for the five models.

Evaluation of mutant fitness data

The mutant fitness data from Deutschbauer et al. [3] contains 195 experiments with fitness scores for 3,355 knockout mutants. The scores ranged from a minimum value of -24.4 to a maximum value of 7.2. Assuming that mutants with a fitness score above the z-score cutoff is able to grow and those below the cutoff is unable to grow, the overall percentage of mutants that is considered to be unable to grow is shown in Figure S3 below.

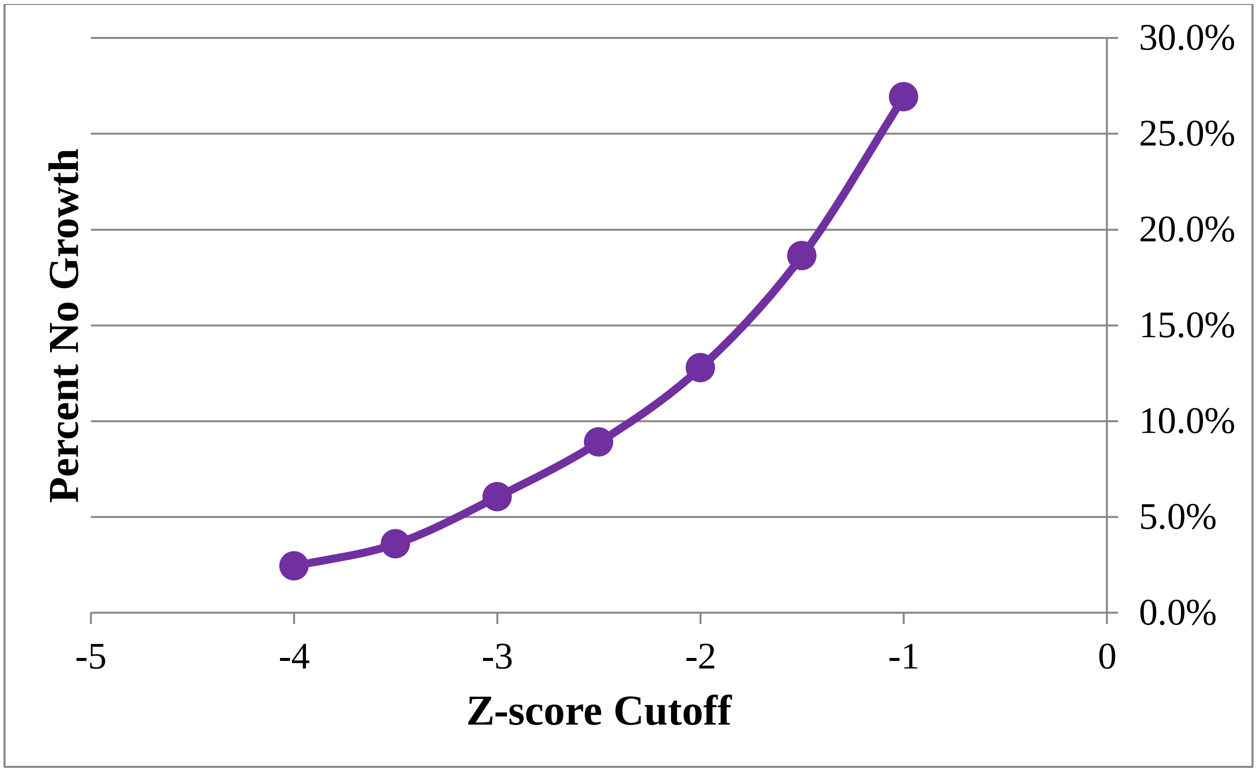

Figure S3. Percent no growth assuming different z-score cutoff for experimental data. Percent of viable mutants unable to grow assuming that mutants with a fitness score below the cutoff is unable to grow.

Types of CONGA results

The five types of CONGA [4] results are defined as:

1. Biomass difference, whereby the two models being compared have different biomass reactions which cause differences in gene essentiality;
2. Metabolic difference, whereby one organism possess an alternative pathway for an essential reaction;
3. Genetic difference, whereby there is a difference in the GPR associations between models for essential reactions;
4. Orthology difference, whereby the same reaction in the two models is catalyzed by non-orthologous genes; and
5. Mixed difference, which represents some combination of the four types of differences above.

First, a biomass difference between models is due to two organisms having different components in their biomass reactions. Here, the deletion of a gene responsible for producing a compound that is needed in only one of the biomass reactions will be lethal only for that organism. For example, because some of the sequenced *Shewanella* strains are missing genes associated with the reactions that produce putrescine (ptrc) and spermidine (spmd), these two compounds were removed from the biomass reaction in the Core model but were kept in the other four models. This caused the deletion of any genes associated with the spermidine biosynthesis pathway to be lethal in each of the four strain-specific models and not the Core model (see Figure S4A). Another type of biomass difference associated with gene deletion sets found by CONGA was caused by differences in lipopolysaccharide (LPS). The five *Shewanella* models use slightly different LPS biosynthesis reactions. This affects the LPS component of the biomass reaction thus resulting in a different biomass reaction definition among the five *Shewanella* models. See "Biomass composition and lipopolysaccharide reactions" section below. One way to tell if the gene deletion set identified by CONGA is due to a biomass difference is to use the SMILEY algorithm [5], which can find alternative reaction(s) that allows one organism to grow and not the other. If SMILEY fails to identify the reaction(s) that allows the non-lethal organism to grow, then the given gene deletion set identified by CONGA is probably due to some difference in the biomass reaction.

A second type of CONGA result is due to metabolic differences between models, and is typically the result of one organism having an alternative pathway for the production of an essential metabolite. This type of CONGA result can easily be identified from the CONGA output by looking for gene deletion sets that resulted in the same reactions being deleted from both organisms. From there, the SMILEY algorithm can once again identify the alternative reactions that if transferred from the non-lethal network to the lethal network would enable the latter to predict growth. For example, the deletion of the gene associated with prephenate dehydrogenase (PPND) was found to be lethal only in the Core model. SMILEY suggested that the reaction missing in the Core model that is present and enables a growth prediction in all the other 4 *Shewanella* models was dihydropteridine reductase (DHPRx). Thus, we investigated the networks surrounding PPND and DHPRx and found that the reason the removal of the PPND reaction was lethal in the Core model was due to the loss of the ability to produce tyrosine. Since

the other models have DHPRx, prephenate (pphn) can be converted first to phenylpyruvate (phpyr), followed by phenylalanine (phe-L) and finally to tyrosine (tyr-L) (see Figure S4B).

CONGA can also identify a genetic difference between models, defined as a difference in the GPR association for a reaction(s) where one organism has more isozymes for a particular reaction. Take the asparagine synthase (ASNS1) reaction for example; the iMR1_799 model has two isozymes that catalyze this reaction, WbpQ (SO3175) and AsnB (SO2767), while all the other models only have AsnB. Since ASNS1 is involved in the synthesis of asparagine, which is an essential metabolite, when the gene associated with AsnB (SO2767) is deleted, only the iMR1_799 model is capable of using WbpQ (SO3175) to produce asparagine and thus predicts growth for the AsnB knockout (see Figure S4C). This type of CONGA result can easily be identified from the CONGA output by looking for gene deletion sets that result in different number of reactions being deleted from the two models being compared.

Other examples of genetic differences were found for two reactions whose associated genes were identified by CONGA that resulted in the Core model being able to grow. The first reaction (G5SD) is catalyzed by glutamate-5-semialdehyde dehydrogenase and converts L-glutamate 5-phosphate (glu5p) to L-glutamate 5-semialdehyde (glu5sa). Every Shewanella strain studied has a ProA enzyme that catalyzes the G5SD reaction; however, there are two different orthologs that encode the ProA enzyme, and some strains have only one of the two. Since the Core model represents the collective group and G5SD is an essential reaction (meaning it has to stay in the Core model), the two genes associated with ProA were encoded as isozymes in the Core model. This led CONGA to predict that the Core model was able to grow while the other models with only one of the two genes cannot when their only ProA ortholog was deleted (see Figure S4D). A similar scenario occurred with the acetylornithine deacetylase (ACODA) and inorganic diphosphatase (PPA) reactions.

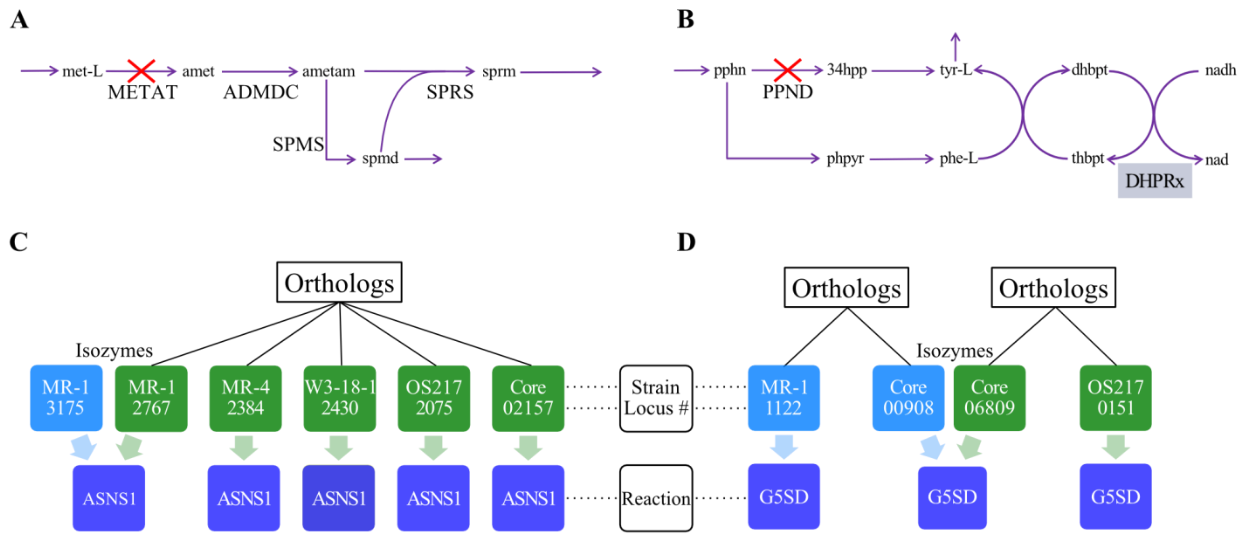

Figure S4. Types of CONGA results identified in this study. Examples shown here were taken from the single deletion case under aerobic pyruvate conditions. (A) Biomass difference – deletion of gene associated with METAT is lethal in all models except Core. Alternatively, the genes associated with ADMDC or (SPMS and SPRS) may be deleted. (B) Metabolic difference – deletion of gene associated with PPND is lethal only in Core due to missing gene associated with DHPRx (shaded). (C) Genetic difference – deletion of gene MR1_2767 orthologs are lethal in all models except *i*MR1_799. (D) Genetic difference due to Core specific isozymes – deletion of gene Core_00908 is lethal only in *i*MR1_799 and deletion of gene Core_06809 is lethal only in *i*OS217_672. amet, S-adenosyl-L-methionine; ametam, S-adenosylmethioninamine; spmd, spermidine; sprm, spermine; pphn, prephenate; 34hpp, 3-(4-hydroxyphenyl)pyruvate; tyr-L, L-tyrosine; dhbpt, 6,7-dihydrobiopterin; phphy, phenylpyruvate; thbpt, tetrahydrobiopterin; METAT, methionine adenosyltransferase; ADMDC, adenosylmethionine decarboxylase; SPMS, spermidine synthase; SPRS, spermine synthase; PPND, prephenate dehydrogenase; DHPRx, dihydropteridine reductase; ASNS1, asparagine synthase; G5SD, glutamate-5-semialdehyde dehydrogenase.

CONGA results summary for double deletion

Just as we have seen for the single deletion case, the Core model is clearly the least robust of the five models under aerobic condition due to the high number of pairs of lethal genes (see Figure S5 in below). This is followed by *i*OS217_672, *i*MR4_812, *i*MR1_799, and *i*W3181_789. The total number of pairs of lethal genes found is much lower than the scenario where only one gene

is deleted, with the exception of the L-alanine plus nitrate condition. The total number of pairs of lethal genes for *i*W3181_789 and *i*OS217_672 is large mainly due to the number of subunit combinations for the reactions involved. For example, for *i*W3181_789, all 16 pairs of lethal genes which are due to a metabolic difference when compared to *i*MR4_812 map to nitrate reductase reactions NTR4 and NTR5. Both of these reactions are catalyzed by two isozymes whereby each isozyme consists of either four or five subunits. For these cases, *i*MR4_812 was predicted to be able to grow without NTR4 and NTR5 because it has a nitrite transporter (NO3t7) and an intracellular nitrate reductase (NTR4B) that are both missing in *i*W3181_789.

There are cases where the deletion of one gene may be due to both genetic and metabolic differences depending on which models it is being compared to. For example, the deletion of MR4_2874 is lethal in *i*MR4_812 but the deletion of the orthologous *i*MR1_799 gene is not because *i*MR1_799 has two isozymes for the same reaction, thus, a genetic difference. On the other hand, the deletion of MR4_2874 is picked up by CONGA as being lethal in *i*MR4_812 and non-lethal in the Core model due to a biomass difference.

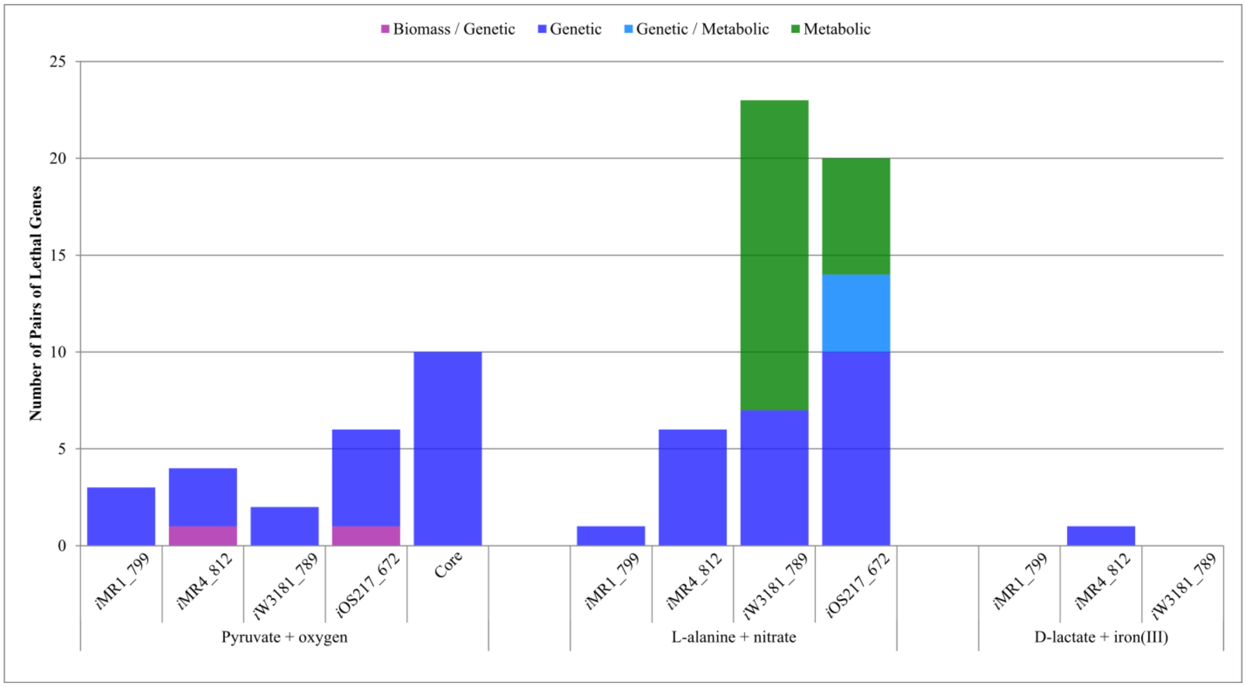

Figure S5. CONGA results summary for double deletion case under all three conditions. Shown here are the numbers of unique pairs of lethal genes identified by CONGA for each model that are not lethal in at least one other model. The numbers are split into the three types of

differences; biomass, metabolic, and genetic difference. Cases where a lethal gene is caused by different types of differences depending on the models being compared are shown.

References

1. Pinchuk GE, Hill EA, Geydebrekht OV, De Ingeniis J, Zhang X, Osterman A, Scott JH, Reed SB, Romine MF, Konopka AE, Beliaev AS, Fredrickson JK, Reed JL: **Constraint-based model of Shewanella oneidensis MR-1 metabolism: a tool for data analysis and hypothesis generation.** *PLoS Comput Biol* 2010, **6:**e1000822.
2. Vinogradov E, Korenevsky A, Beveridge TJ: **The structure of the rough-type lipopolysaccharide from Shewanella oneidensis MR-1, containing 8-amino-8-deoxy-Kdo and an open-chain form of 2-acetamido-2-deoxy-D-galactose**. *Carbohydrate research* 2003, **338**(19):1991-1997.
3. Deutschbauer A, Price MN, Wetmore KM, Shao WJ, Baumohl JK, Xu ZC, Nguyen M, Tamse R, Davis RW, Arkin AP: **Evidence-Based Annotation of Gene Function in Shewanella oneidensis MR-1 Using Genome-Wide Fitness Profiling across 121 Conditions**. *Plos Genetics* 2011, **7**(11).
4. Hamilton JJ, Reed JL: **Identification of Functional Differences in Metabolic Networks Using Comparative Genomics and Constraint-Based Models**. *PLoS One* 2012, **7**(4).
5. Reed JL, Patel TR, Chen KH, Joyce AR, Applebee MK, Herring CD, Bui OT, Knight EM, Fong SS, Palsson BO: **Systems approach to refining genome annotation**. *P Natl Acad Sci USA* 2006, **103**(46):17480-17484.
